# Comparison of Carcass Composition and Meat Quality of the Guinea Fowl (*Numida meleagris*) and the Common Pheasant (*Phasianus colchicus* L.)

**DOI:** 10.3390/ani16060908

**Published:** 2026-03-13

**Authors:** Marcin Wegner, Dariusz Kokoszyński, Marek Kotowicz, Monika Lubawińska

**Affiliations:** 1 Boehringer-Ingelheim, 00-728 Warsaw, Poland; 2 Department of Animal Breeding and Nutrition, Bydgoszcz University of Science and Technology, 85-084 Bydgoszcz, Poland; monikalubawinska01@wp.pl; 3 Department of Meat Science, West Pomeranian University of Technology, 71-550 Szczecin, Poland; marek.kotowicz@zut.edu.pl

**Keywords:** carcass composition, common pheasant, guinea fowl, meat quality

## Abstract

This study aimed to compare carcass composition and selected meat quality traits of guinea fowl (*Numida meleagris*) and common pheasant (*Phasianus colchicus* L.) reared under the production conditions applied in this experiment. The study material consisted of 32 birds, including 16 male common pheasants and 16 male guinea fowl, all slaughtered at 13 weeks of age. The analysis revealed significant differences (*p* < 0.05) between the two groups in carcass composition and several meat quality parameters. Under the given rearing conditions, guinea fowl were characterized by higher body and carcass weight, as well as weights of individual carcass components, compared to pheasants. They also showed higher carcass yield and greater proportions of certain elements, including leg muscles, skin with subcutaneous fat, and wings, whereas pheasants exhibited a higher proportion of breast muscles and neck. Guinea fowl had higher absolute masses of meat, fat, and bones, but a lower meat-to-fat ratio. No significant differences between groups were observed for the meat-and-fat-to-bone ratio or the meat-to-bone ratio. The highest protein content was recorded in the breast muscles of pheasants (27.1%), while the lowest was found in the leg muscle of guinea fowl (22.1%). Differences between the groups were also observed in intramuscular fat and water content in both breast and leg muscles, as well as in collagen content in the breast muscle. Regardless of group, breast muscles were characterized by higher protein content and lower fat and collagen levels than leg muscles. Differences were further noted in electrical conductivity (EC) and the a* and b* color parameters in both muscle types. Breast muscles exhibited lower pH and a* values but higher EC and L* values than leg muscles in both groups. Textural traits of the breast muscles, including cohesiveness, springiness, and chewiness, were higher in guinea fowl, whereas hardness and Warner–Bratzler shear force (WB) were lower compared to pheasants. However, these differences should be interpreted with caution, as the birds were reared under different feeding and management systems, which may have contributed to the observed variation. Overall, the results provide comparative data on carcass composition and meat quality of guinea fowl and pheasants under the studied production conditions. These findings may serve as a basis for further controlled studies designed to more clearly isolate species effects and to evaluate their potential relevance for poultry production.

## Simple Summary

This study compared the carcass composition and meat quality of guinea fowl and common pheasants. Guinea fowl had higher body and carcass weight, more leg muscles, fat, and bones, and higher carcass yield than pheasants, while pheasants had more breast muscle content. Breast muscles in both species had more protein and less fat and collagen than leg muscles. Guinea fowl breast meat was softer, more elastic, and cohesive, whereas pheasant meat was firmer. These results highlight differences in meat composition and quality between the two species and provide useful information for poultry production and meat evaluation.

## 1. Introduction

Guinea fowl (*Numida meleagris*) originate from wild African populations; however, they are currently distributed across all continents and are most commonly reared in free-range systems [1]. This species is primarily used for meat and egg production [2], although in Poland, guinea fowl are rarely exploited for these purposes and are mainly kept as ornamental birds. In contrast, pheasants were originally introduced to Europe from Asia as a game species. Owing to the development of aviary breeding, these birds have become permanently integrated into local ecosystems, and are now widespread throughout Europe, as well as having been introduced to North America [3].

On a global scale, the largest producers of guinea fowl eggs and meat are European countries such as France, Italy, Belgium, and Hungary [2]. In contrast, in Africa, guinea fowl play a significant role in poultry production, ranking second in economic importance after chickens [4]. In Poland, guinea fowl meat represents only a marginal segment of the consumer market, largely due to the limited availability of carcasses in retail outlets. In recent years, however, guinea fowl carcasses have increasingly appeared at local markets and fairs, although low consumer interest in this poultry species continues to limit demand. Similarly, under Polish conditions, pheasants are perceived primarily as game birds. During the 2021–2022 hunting season, approximately 53,000 pheasants were harvested by hunters [5]. Across Europe, pheasant meat originates from hunting and specialized farm production systems [6].

The body weight of adult pheasants is variable, ranging from 900 to 1900 g, depending on the breed, age, sex, and rearing conditions [7]. Guinea fowls, on the other hand, weigh less than 1 kg at 8 weeks of age [4], while other reports indicate that at 65–91 days their body weight may range from 1.4 to 2.1 kg [8]. In both species, determining the optimal slaughter age and slaughter yield remains an important production issue, which has been the subject of numerous studies. Research on the slaughter performance of guinea fowl and pheasants indicates that both species require careful determination of the optimal slaughter age to achieve the best production results. Yamak et al. [4] reported that guinea fowl reach a slaughter yield of approximately 70% at 8 weeks of age. According to Nikolova et al. [9], the slaughter performance of guinea fowl is strongly age-dependent, with birds most commonly slaughtered at 14, 16, or 20 weeks of age. Birds kept beyond 30 weeks achieved very high slaughter yields, ranging from 85.6% to 87.4%, with the proportion of breast muscles differing by sex, 27.1% in males and 28.3% in females. Similarly, numerous studies have been conducted to determine the optimal slaughter age for pheasants. The most favorable time for slaughter is considered to be when birds have reached full plumage and a body weight of approximately 1 kg [10]. Depending on the rearing system, pheasants reach the slaughter age between 12 and 20 weeks of life [11]. The slaughter yield of pheasants ranges from 70.7% to 73.4%, which is higher than that of partridges (49.0–56.8%) and ducks (67.0%) and comparable to the slaughter yield of broiler chickens (70.5–73.3%) [3,11].

The basic meat quality parameters that determine its technological properties include pH, water-holding capacity, color, basic chemical composition, texture, and weight loss during thermal processing [3,8]. Among these traits, meat color is of particular importance, as it is one of the first characteristics assessed by consumers and serves as a key indicator of meat quality, largely dependent on its acidity [2]. Guinea fowl meat is distinguished by its high nutritional value due to its rich content of high-quality protein, essential unsaturated fatty acids, minerals including iron, and vitamins, particularly niacin. At the same time, it has lower cholesterol content compared to chicken meat, which enhances its dietary appeal [12]. Consumers appreciate guinea fowl meat not only for its health benefits but also for its distinctive sensory properties, with flavor and aroma reminiscent of wild game [8]. Similarly, pheasant meat is recognized as a product of high nutritional quality. It is a valuable source of B vitamins (B_6_, B_12_, niacin), high-quality protein, and microelements such as selenium and phosphorus. Pheasant meat is characterized by low fat content and the presence of essential unsaturated fatty acids [11,13,14]. The cholesterol content in pheasant breast and leg muscles ranges from 29 to 71.3 mg per 100 g of meat, which is comparable to the levels found in broiler chicken meat [11].

This study aimed to compare two bird species—the common pheasant (*Phasianus colchicus* L.) and the guinea fowl (*Numida meleagris*)—in terms of body weight, carcass weight, carcass composition, and selected meat quality parameters, including both physicochemical properties and texture characteristics. The research hypothesis assumed that significant differences would exist between the two species in both carcass composition and the examined meat quality traits.

## 2. Materials and Methods

The experiment followed the applicable regulations on protecting animals used for scientific or educational purposes. The study and methods were carried out after obtaining the Experimental Unit’s opinion at the Bydgoszcz University of Science and Technology (No. 17/2010). The ethics committee of Bydgoszcz University of Science and Technology approved the experimental protocols presented in this study.

### 2.1. Materials

This study was conducted on 16 male common pheasants (*Phasianus colchicus* L.) and 16 male guinea fowl (*Numida meleagris*) at 13 weeks of age. The pheasants were randomly selected from an aviary housing 2500 birds and were purchased from a commercial pheasant and partridge farm, “Pheasant and Partridge Farm” in Klon, Poland. The guinea fowl were randomly selected from a poultry house containing 1000 birds and purchased from a commercial poultry farm.

According to the information provided by the breeder, until 3 weeks of age, the pheasants were kept in an indoor facility under controlled environmental conditions, with the floor covered with wood shavings. During this period, the temperature in the rearing facility under the heat lamps was 33 °C on the first day of life and was gradually reduced by approximately 0.5 °C per day until the second week of age (26 °C). From day 1 to day 10 of life, a continuous 24-h lighting program was applied, whereas from day 11 to day 21, 20 h of light and 4 h of darkness were used. The relative air humidity in the poultry house was maintained at 55–60%. In the initial period, the birds were fed a Pheasant 1 diet containing 25.5% crude protein and 2.800 kcal of metabolizable energy (ME). From weeks 4 to 12 of rearing, the pheasants were housed in an outdoor aviary at a stocking density of 3 birds per 1 m^2^. In the final rearing stage (from 13 weeks of age), the birds were transferred to a large winter aviary planted with spring barley and rapeseed, with a sand-covered floor in both aviaries. During weeks 4 to 12, the birds were fed the Pheasant 2 diet containing 22.1% crude protein and 2.750 kcal ME. In the final rearing stage, the pheasants received the Pheasant 3 diet, containing 18.2% crude protein and 2.750 kcal ME, supplemented with whole maize grains. Throughout the rearing period, the pheasants had ad libitum access to fresh drinking water.

The guinea fowl were kept in a poultry house with controlled environmental conditions, including 60–70% humidity and a temperature gradually reduced from 35 °C on the first day of life to 20–22 °C by the eighth week. During the first weeks of life, the birds were exposed to 24 h/day lighting, which was gradually reduced to 20 h/day by the fourth week. Light intensity was further reduced to 15–20 lx by the eighth week. The stocking density in the poultry house was 8 birds/m^2^, and the floor was covered with chopped straw. The birds were fed complete diets containing 24% protein and 2.850 kcal ME until 3 weeks of age, 22% protein and 2.800 kcal ME from 4 to 8 weeks, and 18% protein and 2.700 kcal ME from 8 weeks onward.

### 2.2. Carcass Analysis

At 13 weeks of age, randomly selected male common pheasants (16 birds) and male guinea fowl (16 birds) were weighed using a hook scale WGJ-R (Jofatan, Kraków, Poland) with an accuracy of 1 g to determine body weight. The birds were then manually slaughtered. Stunning was performed by a blunt blow, followed by severing the blood vessels in the neck to allow exsanguination. Subsequently, the birds were scalded, plucked, and eviscerated. The eviscerated carcasses, including the neck, were chilled in a refrigeration cabinet (Hendi, Gadki, Poland) at 2 °C for 18 h. After chilling, the weight of each carcass was recorded, and the carcasses were dissected using a simplified method described by Ziołecki and Doruchowski [15]. The components obtained during carcass dissection—breast muscles, leg muscles, neck, wings, skin with subcutaneous fat, and remaining parts—were weighed separately on an electronic scale WLC 6/12/F1/R (Radwag, Radom, Poland) with an accuracy of 0.1 g. The percentage contribution of each carcass component relative to the weight of the chilled carcass with neck was then calculated. After dissection, the muscle, fat, and bones were weighed using the same scale. Based on these measurements, the mass of muscles (breast and leg), fat (skin with subcutaneous fat), and bones (neck, wings, and remainders) was determined, and the following ratios were calculated: meat-to-fat ratio, meat-to-bone ratio, and the combined meat and fat to bone ratio.

### 2.3. Physicochemical Analyses

At 24 h post-mortem, the pH and electrical conductivity (EC) of the superficial breast muscles and leg muscles (pH_24_ and EC_24_) were measured. The pH was determined using a CX-701 pH meter with a glass electrode inserted into a steel probe (Elmetron, Zabrze, Poland). Before measurements, the device was calibrated with standard buffer solutions at pH 7.0 and 4.0. The results were read from the LCD with an accuracy of 0.01. Electrical conductivity (EC_24_) of the meat was measured using an LF-Star CPU conductometer (Ingenieurbüro R. Matthäus, Nobitz, Germany) with an accuracy of 0.1 mS/cm. The electrode was inserted into the breast and leg muscles at a 90° angle relative to the direction of muscle fibers. The color of the breast and leg muscles was also evaluated using a MINOLTA CR-400 colorimeter (Konica Minolta, Tokyo, Japan) according to the CIE system, recording L* (lightness), a* (redness), and b* (yellowness) values.

### 2.4. Basic Chemical Composition

A total of 90 g of breast meat and 90 g of leg meat from each carcass were collected, and each sample was minced using an electric meat grinder (Zelmer, Rzeszów, Poland). Subsequently, the content of protein, fat, collagen, and water in the breast and leg muscles was determined using near-infrared spectrometry with calibration based on artificial neural networks (ANN), in accordance with the PN-A-82109 standard [16], using the FoodScan™ Meat Analyzer (FoodScan, Hillerød, Denmark).

### 2.5. Texture Analysis

Texture analysis was performed on the breast muscle (*pectoralis major*), which was cooked to 70.2 °C and then cooled to approximately 12 °C. The texture was evaluated according to the procedures for texture profile analysis (TPA) on meat described by Bourne [17], using a Stable Micro Systems TA.XT Plus device (Stable Micro Systems, Godalming, UK), employing both the TPA test and the Warner–Bratzler (WB) shear test. In the TPA test, a cylindrical probe with a diameter of 0.61 cm was compressed twice parallel to the muscle fiber direction to 80% of its original height (16 mm). A head speed of 50 mm/min and a 50 N load cell were used. The force deformation curve obtained from the TPA test was used to calculate the hardness, cohesiveness, springiness, and chewiness of the meat. For the WB test, muscle samples measuring approximately 10 × 10 × 20 mm were cut and sheared using a triangular blade oriented perpendicular to the muscle fibers, with a traverse speed of 50 mm/min and a 500 N load cell. Both the TPA and WB tests were repeated three times for each sample.

### 2.6. Statistical Analysis

The collected numerical data on carcass traits (body weight, carcass weight, and the mass and proportion of individual components) and muscle traits (chemical composition, intramuscular fat, water and collagen content, pH, electrical conductivity, color, and texture) were analyzed using standard methods of mathematical statistics. For carcass traits, a one-way analysis of variance (one-way ANOVA) was applied with species (guinea fowl vs. pheasant) as a fixed factor, according to the linear model: Yik = μ + ai + eik, where Yik—value of the analyzed trait, μ—the overall mean for the tested trait, ai—effect of i-th group, eik—random error. For muscle traits, the analysis was performed separately for the breast muscles (BM) and leg muscles (LM), also with species as a fixed factor. The interaction between species × muscle type was not tested because the analyses were performed separately for each muscle in accordance with the study design. Differences between means were evaluated using the *t*-test at a significance level of *p* < 0.05. Results are presented as means ± standard deviation (SD), with a sample size of *n* = 16 for each group. The statistical characteristics of the studied features were calculated using SAS software (SAS Intitute, Cary, NC, USA), version 9.4.

## 3. Results

Analysis of carcass composition (Table 1) revealed significant differences (*p* < 0.05) in body weight and carcass traits between grey guinea fowl and common pheasants. The average body weight of guinea fowl was 1672.9 g, nearly twice that of pheasants (911.6 g) (*p* < 0.001).

These differences were reflected in carcass weight, breast muscle mass, leg muscle mass, skin with subcutaneous fat, neck, wings, remaining parts, and dressing percentage (*p* < 0.001). The percentage composition of carcass components shows significant differences (*p* < 0.05) between the compared species. The proportion of breast muscles in the carcass was significantly higher in pheasants (25.3%) than in guinea fowl (22.6%) (*p* < 0.001). The opposite trend was observed for leg muscles, with their proportion in the carcass being significantly higher in guinea fowl (22.9%) compared to pheasants (20.2%) (*p* = 0.005). Guinea fowl also showed a significantly higher proportion of skin with subcutaneous fat and wings (*p* < 0.001). In contrast, the proportion of the neck in the carcass was significantly higher in pheasants (5.4%) than in guinea fowl (4.7%) (*p* = 0.020). No significant differences were observed between species for the proportion of remaining carcass components (*p* = 0.442).

Analysis of carcass traits (Table 2) revealed significant differences between the compared species in terms of the absolute mass of muscles, bones, and fat, as well as their proportions. The meat mass in guinea fowl carcasses was significantly higher (591.3 g) than in pheasants (309.7 g) (*p* < 0.001). A similar trend was observed for fat mass, which was 107.8 g in guinea fowl and only 40.4 g in pheasants (*p* < 0.001). Bone mass was also significantly higher in guinea fowl (*p* < 0.001). Analysis of the mass ratios showed a significantly lower meat-to-fat ratio in guinea fowl (5.49) compared to pheasants (7.65) (*p* < 0.001), indicating a relatively higher fat content in guinea fowl carcasses. In contrast, no significant differences were observed between species in the meat-and-fat-to-bone ratio or in the meat-to-bone ratio, with similar values in both species (*p* = 0.373, 0.743; respectively).

Table 3 presents the basic chemical composition of breast (BM) and leg (LM) muscles of grey guinea fowl and common pheasants. The analysis revealed a significant effect of both species and muscle type on protein, intramuscular fat, water, and collagen content.

Protein and fat content were significantly higher in both breast and leg muscles of pheasants (*p* < 0.001). The highest protein content was observed in the breast muscle of pheasants (27.1%), while the lowest was in the leg muscles of guinea fowl (22.1%). Additionally, significant differences were found between muscle types regardless of species, with breast muscles exhibiting higher protein content than leg muscles (*p* < 0.001). In turn, intramuscular fat content was significantly higher in leg muscles compared to breast muscles (*p* < 0.001). Water content was significantly higher in guinea fowl than in pheasants, both in breast muscles (*p* < 0.001) and in leg muscles (*p* = 0.018). No significant differences were observed between muscle types in water content (*p* = 0.054). Collagen content did not differ significantly between species in the breast muscle (*p* = 0.376); however, a significant effect was observed in leg muscles (*p* < 0.001) and depending on muscle type (*p* = 0.047). In the leg muscles, collagen content was significantly higher in guinea fowl (1.9%) compared to pheasants (1.4%) and also higher than in breast muscles (1.5%).

Table 4 presents selected physicochemical traits of breast (BM) and leg (LM) muscles of grey guinea fowl and common pheasants. Statistical analysis revealed a significant effect of muscle type on most of the evaluated parameters, as well as a species-dependent variation depending on the specific trait. The pH measured 24 h post-mortem (pH_24_) did not differ significantly between species in either breast or leg muscles (*p* = 0.229, 0.973; respectively). However, significant differences were observed between muscle types regardless of species (*p* < 0.001), with leg muscles exhibiting higher pH values than breast muscles. Electrical conductivity of the muscles (EC_24_) was significantly higher in guinea fowl than in pheasants, both in breast and leg muscles (*p* < 0.001). Moreover, irrespective of species, breast muscles showed significantly higher electrical conductivity compared to leg muscles (*p* < 0.001). Analysis of color parameters revealed a significant effect of muscle type on lightness (L*), with breast muscles being lighter than leg muscles regardless of species (*p* < 0.001). No significant differences between species were observed for breast muscle lightness (*p* = 0.278), whereas leg muscles of guinea fowl exhibited significantly higher lightness compared to pheasants (*p* = 0.011). The a* value (redness) was significantly higher in guinea fowl than in pheasants, both in breast and leg muscles (*p* = 0.016, 0.017, respectively). Additionally, leg muscles exhibited significantly higher a* values compared to breast muscles, regardless of species (*p* < 0.001). The b* value (yellowness) was also significantly higher in guinea fowl than in pheasants, in both breast and leg muscles (*p* = 0.027, 0.031; respectively). However, no significant effect of muscle type on b* was observed regardless of species (*p* = 0.179).

Table 5 presents selected textural traits of the breast muscles of male guinea fowl and common pheasants. Significant differences (*p* < 0.05) were observed between the species for all evaluated parameters. Warner–Bratzler shear force (WB) was significantly lower in guinea fowl (58.1 N) compared to pheasants (100.9 N) (*p* < 0.001). Hardness of the breast muscles was also significantly lower in guinea fowl (18.9 N) than in pheasants (24.0 N) (*p* < 0.001). In contrast, cohesiveness, springiness, and chewiness were higher in guinea fowl compared to pheasants (*p* < 0.001).

## 4. Discussion

Numerous studies have shown that carcass composition, the mass and proportion of individual components, and slaughter yield in poultry depend on factors such as genotype, sex, nutrition, and rearing system [18,19,20]. In the current study, the average body weight of guinea fowl was nearly twice that of pheasants, which was reflected in carcass weight (1302.6 g—guinea fowl vs. 679.9 g—pheasant). Carcass yield of guinea fowl (77.9%) was also significantly higher than that of pheasants (74.6%). This difference in yield is consistent with previous studies [21,22], which showed that guinea fowl exhibit high slaughter performance. However, it should be noted that differences observed between guinea fowl and pheasants may also reflect differences in diet, rearing system, and physical activity, rather than species alone. regardless of age or feeding regimen. Gasparovic et al. [22] report that pheasants are characterized by a high carcass yield of 69.7–73.7% when slaughtered at 16–20 weeks of age. This value is similar to the results obtained for pheasants in our study and is considerably lower than that for guinea fowl. Potentially due to differences in rearing conditions and management practices rather than inherent species effects. The observed higher body weight, carcass weight, and breast and leg muscle mass in guinea fowl compared to pheasants may contribute to better slaughter performance. However, these results are not solely due to species differences, but are also influenced by the rearing system, feed intake, and activity level of the birds, which is consistent with the literature highlighting the musculature development of guinea fowl [19,23,24]. In this work, the proportion of breast muscles was higher in pheasants. Baéza et al. [25] demonstrated that standard guinea fowl at 13.5 weeks of age were heavier than organic birds, whereas the proportion of breast and leg muscles was higher in organically raised birds. Bernacki et al. [20] reported breast muscle proportions of 17–22%, similar to the results obtained in the present study (22.6%), while Zelleke et al. [24] indicated approximately 21.4%, confirming that both breed and rearing system influence carcass structure. Analysis of carcass traits revealed differences between guinea fowl and pheasants in the mass of meat, fat, and bones, as well as in their proportions. These differences reflect the combined effects of species and rearing conditions, and cannot be attributed to species alone.

Analysis of carcass traits revealed significant differences between guinea fowl and pheasants in the mass of meat, fat, and bones, as well as in their proportions. The considerably higher meat mass in guinea fowl carcasses compared to pheasants confirms their greater meat potential, which is consistent with other studies on guinea fowl [23]. Although guinea fowl meat is often described as lean in terms of intramuscular fat content [26,27], total fat mass and distribution can also be influenced by diet, age, sex, and activity level [23]. The lower fat mass in pheasant carcasses aligns with observations that wild gallinaceous birds generally have lower fatness compared to farmed species [28], which in this study may also reflect differences in management and feeding. The lower meat-to-fat ratio in guinea fowl indicates a relatively higher fat content in the carcass, which may positively affect meat juiciness and sensory quality. The lack of differences in the meat-and-fat-to-bone ratios suggests a similar structural composition of carcasses in both species, despite differences in the absolute mass of individual components.

In our study, the analysis of the chemical composition of muscles revealed significant differences between species and muscle types, which is consistent with previous observations in both wild and farmed poultry [19,26]. The chemical composition of pheasant meat was characterized by high protein and low fat content, with breast muscles containing more protein and less moisture than leg muscles. The moisture/protein ratio was relatively low (2.8–3.4), indicating good suitability for processed meat products [29]. The highest protein content (27.1%) was observed in the breast muscles of pheasants, while the lowest was found in the leg muscles of guinea fowl (22.1%). These patterns were similar to previous studies [22,26]. It should be noted that the observed differences between species could also reflect the effects of management, diet, and activity rather than purely species-specific traits. A similar pattern, with higher protein content in breast muscles compared to leg muscles in both guinea fowl and pheasants, has been reported in other studies [22,26]. The higher protein content in breast muscles may be related to their primary function, as these muscles play a key role in short-distance flight or wing movements, requiring a higher proportion of muscle fibers and lower fat content; however, the rearing system and activity level of the birds could also contribute to this difference. The higher protein content in breast muscles may be related to their primary function, as these muscles play a key role in short-distance flight or wing movements, requiring a higher proportion of muscle fibers and lower fat content. Intramuscular fat content was significantly higher in leg muscles than in breast muscles, which aligns with observations from other authors [2,23,26]. Leg muscles serve supportive and locomotory functions, which necessitate energy reserves in the form of fat. Additionally, guinea fowl meat exhibited higher intramuscular fat content in both muscle types compared to pheasants, correlating with the greater total fat mass observed in guinea fowl carcasses. Furthermore, farmed pheasant meat showed high water holding capacity and myofibrillar protein extractability, with slight variations depending on age and muscle cut (*p* < 0.05), which confirms its suitability for processed meat production [29]. Water content analysis showed that guinea fowl muscles had lower water content than pheasants, both in the breast (71.3% vs. 73.8%) and leg muscles (72.8% vs. 73.4%), likely reflecting higher protein and fat concentration as well as management and feeding differences. The literature indicates that lower water content in poultry muscles is often associated with higher protein and fat concentration [28]. The higher water content in pheasant muscles may also result from lower total fat and intramuscular fat content in the carcass. Collagen content was lower in guinea fowl leg muscles (1.4%) compared to pheasants (1.9%), while no differences were observed in breast muscles. These differences may also reflect differences in physical activity, rearing system, or age at slaughter rather than purely species-specific traits. In another study, the authors [30] reported a significantly lower collagen content in the breast muscles of pheasants at 25 weeks of age compared to the results obtained in our study. The higher collagen content in pheasant leg muscles may be attributed to greater locomotory activity and the associated need for stronger muscle fibers [31]. Elevated collagen content also influences meat hardness and texture, which are important parameters for consumer quality.

The pH did not differ significantly between species but was clearly dependent on muscle type. Leg muscles exhibited higher pH values compared to breast muscles. This observation has also been confirmed in other studies [2,32], which is associated with differences in muscle fiber metabolism and the course of postmortem glycolysis. The higher electrical conductivity in guinea fowl muscles could be influenced by management and postmortem handling, in addition to species. The higher electrical conductivity of both breast and leg muscles in guinea fowl compared to pheasants may reflect differences in the intensity of postmortem changes and the integrity of cell membranes, as also reported previously [2]. Color analysis of the muscles showed that leg muscles were darker (lower L*) and more red and yellow (higher a* and b*) compared to breast muscles. These differences should be interpreted cautiously, as they may result from diet, activity, or management conditions, not only species. Similar relationships between muscle type and color parameters (L*, a*, b*) have been described in guinea fowl, where leg muscles exhibited more intense coloration than breast muscles, which was related to higher myoglobin content and differences in muscle fiber composition [2,33]. In another study by Hofbauer, wild pheasants were found to have lower a* values (red) and higher b* values (yellow) in the breast muscles compared to the results obtained in the present study. This may indicate differences in myoglobin content, muscle fiber type, and diet of the wild birds compared to the studied individuals, which affect the intensity and hue of the meat color.

In the conducted study, significant differences were observed in the texture of the breast muscle between guinea fowl and pheasants. The significantly lower Warner–Bratzler (WB) shear force in guinea fowl indicates greater meat tenderness compared to pheasants. Similar observations have been reported in studies on guinea fowl, where this species exhibited lower shear force and higher sensory scores for tenderness compared to other gallinaceous birds, which was associated with lower collagen content [23,26]. Breast muscles of 6-month-old farmed pheasants had lower Warner–Bratzler shear force compared to 17-month-old birds and leg muscles, indicating that age and muscle type significantly influence tenderness [30]. The higher meat hardness observed in pheasants in this study is consistent with reports on game birds, where more intensive physical activity and a higher proportion of oxidative fibers contribute to greater stiffness of muscle structure [33]. At the same time, higher cohesiveness and springiness values in guinea fowl meat may indicate a more uniform and elastic structure of myofibrillar proteins, a trait often attributed to poultry meat with lower intramuscular fat content and favorable muscle fiber arrangement [34]. This means that higher chewiness may reflect a more elastic and cohesive structure without indicating increased meat hardness. Higher chewiness values in guinea fowl meat, despite its greater tenderness as measured by WB, suggest that this meat requires more mechanical energy during mastication, likely due to greater elasticity and cohesiveness of the structure rather than hardness itself. Similar relationships between TPA parameters and shear force have been previously reported in alternative poultry, including guinea fowl and slow-growing chickens [2,34].

This study has several limitations that should be considered when interpreting the results. First, the compared species were reared under different feeding regimes and management systems, which may have influenced carcass composition and meat quality traits. Differences in physical activity levels between groups could also have contributed to the observed variation. Second, only male birds were included in the study, which limits the generalizability of the findings to females. Third, the evaluation was conducted at a single slaughter age (13 weeks), and therefore, age-related changes in carcass and meat quality traits were not assessed. Consequently, the findings should be interpreted as comparative data obtained under specific production conditions rather than as definitive evidence of inherent species differences. Future studies conducted under fully controlled experimental conditions are required to isolate species effects more precisely.

## 5. Conclusions

The current work demonstrated significant differences between guinea fowl and pheasants in carcass composition and selected meat quality traits under the specific rearing conditions applied. Guinea fowl were characterized by higher body and carcass weight, greater leg muscle mass, and higher carcass yield, whereas pheasants showed a higher proportion of breast and neck muscles. Differences were also observed in the chemical composition of muscles (protein, fat, water, collagen), physicochemical parameters (pH, electrical conductivity, meat color), and selected textural properties (hardness, springiness, and chewiness). However, these differences should be interpreted with caution, as the birds were reared under different feeding and management systems, which may have contributed to the observed variation. Therefore, the results reflect differences between the two species under the given production conditions and do not allow unequivocal attribution of the effects solely to the species. The findings provide baseline comparative data on carcass traits and meat quality of guinea fowl and pheasants, which may serve as a foundation for further controlled studies aimed at isolating species effects and evaluating their practical implications in poultry production.

## Figures and Tables

**Table 1:** Body weight, carcass traits, and percentage composition of carcass components of guinea fowl and common pheasant at 13 weeks of age.

Item	Guinea Fowl	Common Pheasant	*p*-Value
Body weight (g)	1672.9 ^a^ ± 170.3	911.6 ^b^ ± 56.8	<0.001
Carcass weight (g)	1302.6 ^a^ ± 134.9	679.9 ^b^ ± 88.2	<0.001
Dressing percentage (%)	77.9 ^a^ ± 1.1	74.6 ^b^ ± 0.8	<0.001
Breast muscles (g)	293.9 ^a^ ± 31.8	172.0 ^b^ ± 24.6	<0.001
Breast muscles in carcass (%)	22.6 ^b^ ± 0.8	25.3 ^a^ ± 2.8	<0.001
Leg muscles (g)	297.8 ^a^ ± 32.9	137.3 ^b^ ± 18.8	<0.001
Leg muscles in carcass (%)	22.9 ^a^ ± 0.8	20.2 ^b^ ± 2.3	0.005
Skin with fat (g)	107.8 ^a^ ± 24.6	40.4 ^b^ ± 4.8	<0.001
Skin with fat in carcass (%)	8.4 ^a^ ± 1.3	5.9 ^b^ ± 0.7	<0.001
Wings (g)	176.8 ^a^ ± 17.2	81.1 ^b^ ± 8.9	<0.001
Wings in carcass (%)	13.6 ^a^ ± 0.2	11.9 ^b^ ± 1.3	<0.001
Neck (g)	61.4 ^a^ ± 6.5	36.9 ^b^ ± 8.5	<0.001
Neck in carcass (%)	4.7 ^b^ ± 0.4	5.4 ^a^ ± 1.4	0.020
Remainders (g)	364.9 ^a^ ± 31.6	212.2 ^b^ ± 28.6	<0.001
Remainders of carcass (%)	28.1 ± 0.7	31.2 ± 4.8	0.442

^a,b^—mean values of traits in rows marked with different letters are statistically significantly different (*p* < 0.05), *n* = 16/species.

**Table 2:** Weight and proportions of meat, fat, and bones in the eviscerated carcass of guinea fowl and common pheasant at 13 weeks of age.

Item	Guinea Fowl	Common Pheasant	*p*-Value
Meat (g)	591.3 ^a^ ± 62.2	309.7 ^b^ ± 37.8	<0.001
Fat (g)	107.8 ^a^ ± 24.6	40.4 ^b^ ± 4.8	<0.001
Bones (g)	598.8 ^a^ ± 49.7	330.2 ^b^ ± 23.0	<0.001
Meat : Fat (x)	5.5 ^b^ ± 0.6	7.6 ^a^ ± 1.2	<0.001
Meat and Fat : Bones (x)	1.2 ± 0.1	1.1 ± 0.2	0.373
Meat : Bones (x)	1.0 ± 0.1	0.9 ± 0.2	0.743

^a,b^—mean values of traits in rows marked with different letters are statistically significantly different (*p* < 0.05), *n* = 16/species.

**Table 3:** Basic chemical composition of meat from male guinea fowl and common pheasants at 13 weeks of age.

Item		Guinea Fowl	Common Pheasant	*p*-Value	
				Species	Muscle Type
Protein, %	BM	24.1 ^by^ ±0.4	27.1 ^ay^ ± 0.3	<0.001	<0.001
	LM	22.1 ^b^ ± 0.4	22.9 ^a^ ± 0.5	<0.001	
Intramuscular fat, %	BM	0.4 ^by^ ± 0.1	0.6 ^ay^ ± 0.1	<0.001	<0.001
	LM	1.8 ^b^ ± 0.1	2.2 ^a^ ± 0.3	<0.001	
Water, %	BM	73.8 ^a^ ± 0.3	71.3 ^b^ ± 0.2	<0.001	0.054
	LM	73.4 ^a^ ± 0.7	72.8 ^b^ ± 0.7	0.018	
Collagen, %	BM	1.5 ^y^ ± 0.1	1.4 ± 0.1	0.376	0.047
	LM	1.9 ^a^ ± 0.2	1.5 ^b^ ± 0.3	<0.001	

Note: Values are expressed as means ± standard deviation, *n* = 16/genotype, BM—breast muscles, LM—leg muscles. ^a,b^ *p* < 0.05—means with different superscripts are statistically different between species. ^y^ *p* < 0.05—statistically significant differences between breast meat and leg meat, regardless of species.

**Table 4:** Selected physicochemical traits of the meat of male grey guinea fowl and common pheasants at 13 weeks of age.

Item		Guinea Fowl	Common Pheasant	*p*-Value	
				Species	Muscle Type
Acidity—pH_24_	BM	6.0 ^y^ ± 0.2	5.9 ^y^ ± 0.2	0.229	<0.001
	LM	6.2 ± 0.2	6.2 ± 0.2	0.973	
Conductivity—EC_24_ (mS/cm)	BM	12.0 ^ay^ ± 0.4	9.7 ^by^ ± 2.2	<0.001	<0.001
	LM	9.3 ^a^ ± 0.8	6.7 ^b^ ± 2.4	<0.001	
Lightness—L*	BM	48.4 ^y^ ± 2.6	46.6 ^y^ ± 5.8	0.278	<0.001
	LM	42.9 ^a^ ± 3.6	39.8 ^b^ ± 2.9	0.011	
Redness—a*	BM	8.7 ^ay^ ± 1.6	7.0 ^by^ ± 2.2	0.016	<0.001
	LM	14.9 ^a^ ± 2.9	12.9 ^b^ ± 1.2	0.017	
Yellowness—b*	BM	5.3 ^a^ ± 2.4	3.6 ^b^ ± 1.7	0.027	0.179
	LM	4.6 ^a^ ± 2.7	2.8 ^b^ ± 1.6	0.031	

Note: Values are expressed as means ± standard deviation, *n* = 16/genotype, BM—breast muscle, LM—leg muscles. ^a,b^ *p* < 0.05—means with different superscripts are statistically different between species. ^y^ *p* < 0.05—statistically significant differences between breast muscles and leg muscles, regardless of species.

**Table 5:** Some textural traits of the breast muscles of male grey guinea fowl and common pheasants at 13 weeks of age.

Item	Guinea Fowl	Common Pheasant	*p*-Value
WB-shear force (N)	58.1 ^b^ ± 16.2	100.9 ^a^ ± 16.8	<0.001
Hardness (N)	18.9 ^b^ ± 1.9	24.0 ^a^ ± 1.3	<0.001
Cohesiveness	0.4 ^a^ ± 0.1	0.3 ^b^ ± 0.1	<0.001
Springiness (cm)	1.5 ^a^ ± 0.1	0.9 ^b^ ± 0.1	<0.001
Chewiness (N × cm)	10.7 ^a^ ± 2.2	4.4 ^b^ ± 1.4	<0.001

Note: Values are expressed as means ± standard deviation, *n* = 16/species. ^a,b^ *p* < 0.05—means with different superscripts are statistically different between species.

## Data Availability

Data will be provided upon request.
